# Comparing body composition between the sweet-liking phenotypes: experimental data, systematic review and individual participant data meta-analysis

**DOI:** 10.1038/s41366-024-01494-7

**Published:** 2024-03-11

**Authors:** Rhiannon Mae Armitage, Vasiliki Iatridi, Martina Sladekova, Martin Richard Yeomans

**Affiliations:** 1 School of Psychology, University of Sussex, Brighton, UK; 2 Department of Sport, Health Sciences and Social Work, Oxford Brookes University, Oxford, UK

**Keywords:** Feeding behaviour, Homeostasis

## Abstract

**Background:**

Legislation aimed at reducing sugar intake assumes that sweet-liking drives overconsumption. However, evidence that a greater liking for sweet taste is associated with unhealthier body size is mixed and complicated by relatively small samples, an overreliance on body mass index (BMI) and lack of classification using sweet-liking phenotypes.

**Methods:**

We first examined body size data in two larger samples with sweet-liking phenotyping: extreme sweet-likers, moderate sweet-likers and sweet-dislikers. Adults (18-34yrs), attended a two-session lab-based experiment involving phenotyping for sweet-liking status and a bioelectrical impedance body composition measurement (Experiment One: *N* = 200; Experiment Two: *N* = 314). Secondly, we conducted an individual participant data (IPD) meta-analysis: systematic searches across four databases identified 5736 potential articles. Of these, 53 papers met our search criteria: a taste assessment that measured liking using sucrose (>13.7% *w/v*), which allowed sweet-liking phenotyping and included either BMI, body fat percentage (BF%), fat-free mass (FFM) or waist-circumference.

**Results:**

A significant effect of sweet-liking phenotype on FFM was found in both Experiment One and Two, with extreme sweet-likers having significantly higher FFM than sweet-dislikers. In Experiment One, sweet-dislikers had a significantly higher BF% than extreme sweet-likers and moderate sweet-likers. However, as these data are from one research group in a young, predominantly westernised population, and the results did not perfectly replicate, we conducted the IPD meta-analyses to further clarify the findings. Robust one-stage IPD meta-analyses of 15 studies controlling for sex revealed no significant differences in BF% (*n* = 1836) or waist-circumference (*n* = 706). For BMI (*n* = 2368), moderate sweet-likers had slightly lower BMI than extreme sweet-likers, who had the highest overall BMI. Most interestingly, for FFM (*n* = 768), moderate sweet-likers and sweet-dislikers showed significantly lower FFM than extreme sweet-likers.

**Conclusion:**

The higher BMI often seen in sweet-likers may be due to a larger FFM and questions the simple model where sweet liking alone is a risk factor for obesity.

## Introduction

Consumption of high-fat sugar diets is a significant factor in the aetiology of obesity and subsequent non-communicable diseases, including cardiovascular disease and type-2 diabetes [1]. Globally, obesity has nearly tripled since 1975 [2], and with rates of obesity and the associated disease burden rising annually worldwide [1], obesity is now predicted to surpass smoking as the leading cause of preventable deaths [3] and in England and Scotland it already has [4]. Therefore, it is becoming more important to better understand the myriad of contributory factors to obesity and why regulation mechanisms allow body weight to increase in some but not all individuals.

As excess body weight arises from sustained positive energy balance [5], the role of food choice and intake are central to understanding the multifactorial nature of obesity [6]. Although genetic factors [7] and changes in the environment significantly contribute to this rapid upward trend in obesity rates e.g., [8–11], individual differences in taste hedonics may increase susceptibility to consume the energy-dense, high-fat sugar and nutrient-poor food and beverages found in ‘westernised diets’ and could therefore play a role in the obesity epidemic e.g., [6, 12, 13]. Taste hedonics have important influences on eating behaviour, informing food preference, selection and, consequently, nutritional intake and health [14]. Therefore, a greater understanding of individual differences in hedonic responses to sweet taste may provide insights into predispositions to diet-related health outcomes to better support public health strategies to prevent obesity and non-communicable diseases [15, 16].

**Fig. 1:**
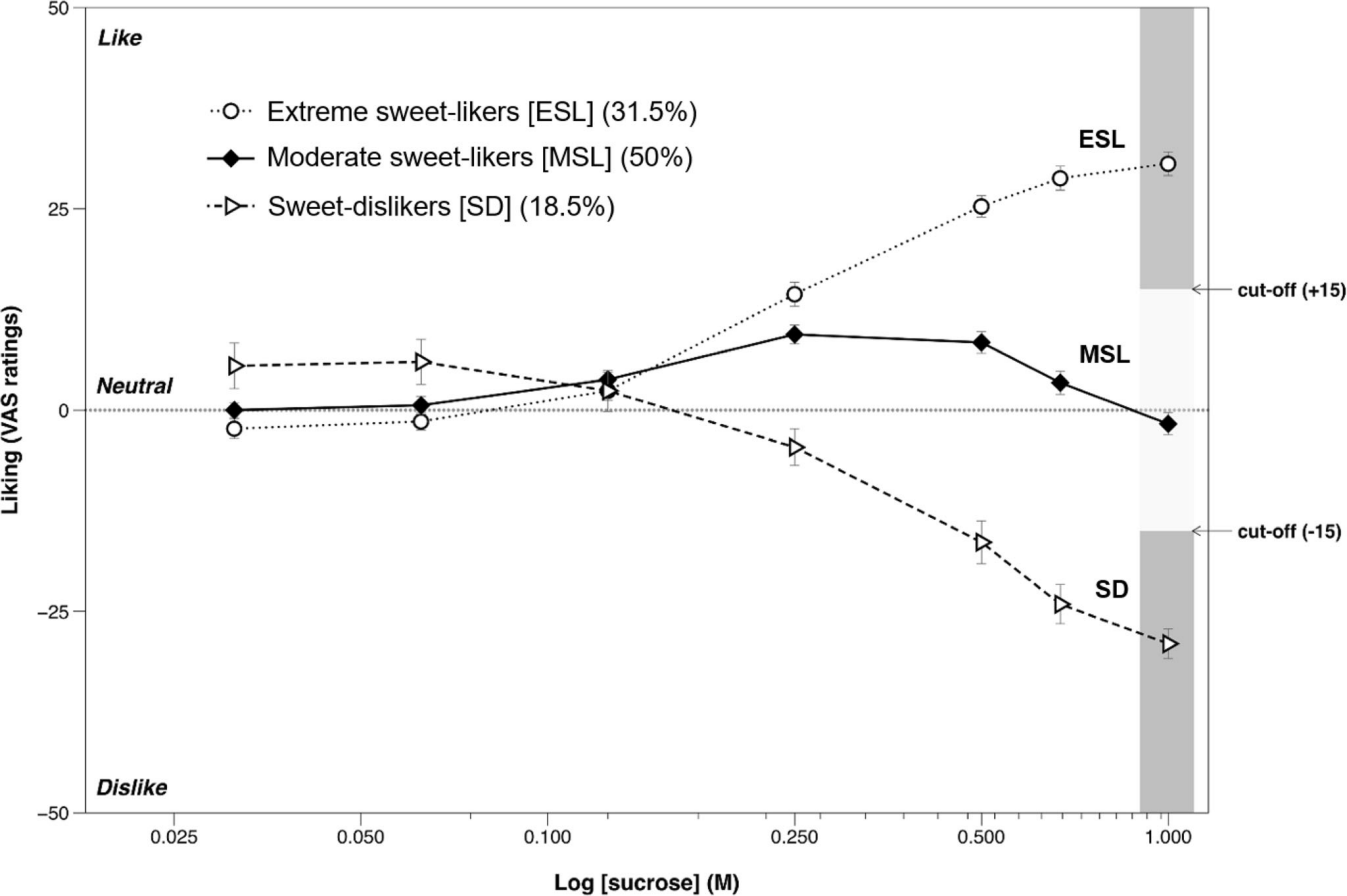
Liking patterns for the three sweet-liking phenotypes defined by hierarchal cluster analysis. Modified with permission from [23], this figure shows the differential liking patterns for the three sweet-liking phenotypes as categorised by hierarchal cluster analysis: Extreme sweet-likers (ESL), whose liking increased with sweetness intensity; Moderate sweet-likers (MSL), whose liking peaked at around 0.25 M sucrose before decreasing; and sweet-dislikers (SD), whose liking decreased with sweetness intensity. See [23] for analysis of the sensitivity and specificity scores in deciding phenotype cut-offs dependent on sucrose concentration.

Long considered an innate preference e.g., [17–19], research from a motivational standpoint has focused on the universality of sweet liking e.g., [20, 21], however, a growing body of research over the last 50 years has shown that sweet taste is not as universally liked as once thought. Historically a dichotomous response pattern favoured research discriminating between those who either like sweet taste (sweet-likers) or dislike sweet taste (sweet-dislikers). However, these often relied on arbitrary cut-offs to distinguish sweet-liking phenotypes, whereas a series of recent studies using more advanced statistical analyses to categorise sweet-liking phenotypes strongly suggest three distinct hedonic responses to sweet taste across adult populations in Europe, North America and Asia e.g., [22–27]: (see Fig. 1). This approach statistically supports the visual interpretation of the three sweet-liking phenotypes noted in the seminal work of Pangborn [28]. These are classified as extreme sweet-likers, where liking increases with sweetness intensity; moderate sweet-likers, who like moderate but not intense sweetness; and sweet-dislikers, who show increasing dislike as sweetness increases.

As sugar consumption and liking for sweet taste is so widely intertwined with obesity in popular media e.g., [29–33], it would be expected that those who most enjoy sweet tastes (i.e., extreme sweet-likers) would have the worst anthropometric profiles (e.g., higher BMI, waist circumference and total body fat). However, while high sugar intake is considered more detrimental than beneficial for health [34], the evidence supporting this widespread belief that high liking is related to an unhealthier body size mixed. For example, only one study to our knowledge has reported higher BMI being associated with greater sweet liking [35], whilst several studies reported lower liking for sweetness in individuals with obesity e.g., [36–43] and many non-significant relationships e.g., [44–46]. However, earlier studies did not consider the impact of sweet-liking phenotypes and predominantly only measured BMI, which is a poor indicator of body fat [47] and cannot distinguish between body tissues; therefore, people without excess adiposity can be categorised as being overweight or obese, particularly for values below 30 kg/m^2^ [48]. Interestingly, studies which included participants with a broader BMI range and who used sweet-liking phenotyping found participants with obesity are more frequently classified as sweet-dislikers than extreme sweet-likers e.g., [37, 39–43].

Sweet-liking research investigating broader indices of obesity beyond BMI, such as waist/hip circumference, skinfold measurements, and body composition analysis, is relatively limited. We are only aware of three studies that included body fat measurements using the traditional dichotomous phenotyping, where moderate sweet-likers are misclassified into the two extreme groups. However, these results were mixed: one study found significant differences, with sweet-dislikers having higher body fat percentage (BF%) [43], while another study reported no differences [42], and the last showed a non-significant trend for sweet-likers to have a greater BF% [26]. These findings were recently extended using the three-phenotype model [22, 49]. One study measuring BMI and BF% in a large museum-based science project found no differences [8], although the other, which also included free-fat mass (FFM) in a younger sample, suggested sweet-dislikers had higher fat-mass, while extreme sweet-likers tended to have higher FFM [49]. However, as we identified in a recent review [50], most previous research had relatively small samples, an overreliance on BMI and had not used sweet-liking phenotyping. Thus, given the growing concerns that an abundance of sweet-tasting foods and beverages drive obesity, those findings investigating the relationship between sweet-liking and body size differences need substantiating.

Here, we aim to address these issues, first by testing further the results reported in [49] in two stronger-powered datasets and, secondly, by conducting an independent participant data (IPD) meta-analysis of all available datasets, which included body composition data and allow sweet-liking phenotyping to standardise categorisation, to provide deeper insights into the role of sweet taste liking as a driver of obesity.

## Experiment One

### Methods

#### Participants

Experiment One recruited 286 adults, aged 18–34, from the University of Sussex and local area (Brighton, UK) to participate in a two-session lab-based study advertised as ‘Taste, Personality and Body Composition Study’. Given the limited data on taste hedonics and body composition, earlier studies have determined it is not yet possible to accurately determine effect sizes [26, 51]. Therefore, sample size was determined from earlier studies between sweet-liking and body composition using wider indices beyond BMI [50], ensuring at least 30 participants in each phenotype to detect differences in FFM [49]. To qualify, participants were required to be free of medication (other than oral contraceptives), non-smokers (fewer than five cigarettes a week), and have no history of diagnosed eating disorders or diabetes. In addition, women needed to report a regular menstrual cycle. Individuals currently dieting or suffering from a respiratory illness were excluded. Written informed consent was obtained on arrival at the research facilities. However, participants remained naive to the true aims of the study until being debriefed after completing all tasks. Testing procedures were approved by The University of Sussex Science and Technology Cross-Schools Research Ethics Committee (ER/MARTIN/12) and followed the guidelines of the Declaration of Helsinki.

#### Taste stimuli

Three water-based taste stimuli were used: a 1.0 M sucrose solution (342.3 g wt/vol) to determine sweet-liking phenotype status alongside two controls: mineral water (0 M) and a 0.125 M sucrose solution (42.8 g wt/vol). These were made weekly using a volumetric flask by dissolving food-grade sugar in mineral water, kept in the fridge (4 °C) and brought to room temperature (22 °C) before testing.

#### Rating scales

Participants evaluated the taste stimuli for liking and intensity. Liking: (‘How much did you like Sample X?’) was assessed using a horizontal visual analogue scale (VAS) end-anchored with “dislike extremely” ( − 50) and “like extremely” ( + 50); and intensity: (‘How intense/sweet/sour/bitter was Sample X?’) using a vertical Generalised Labelled Magnitude Scale (gLMS) ranging from “no sensation” (0) to “strongest imaginable sensation of any kind” [52]. Before participants taste the solutions, training on the two rating scales was provided to ensure within and between participant consistency: for liking, multiple VAS using non-food items; for intensity, a gLMS using non-tested visual and sound sensory stimuli [53]. All ratings were collected using the Sussex Ingestion Pattern Monitor (SIPM version 2.0.13, University of Sussex, Falmer, UK).

#### Procedure

Both sessions were held in the Human Psychopharmacology Lab at the University of Sussex within seven days of each other. The first 50-minute session involved scale training and phenotyping for sweet-liking status followed by a memory task and a series of questionnaires on Qualtrics (Qualtrics, Provo UT, USA: not analysed in this paper) held between 10:00-14:00. The second 20-minute session was an anthropometric assessment conducted between 08:00–11:00, after which participants were debriefed.

##### Session 1: sweet-liking phenotyping and demographics

Participants were required to refrain from eating and drinking flavoured beverages, smoking, chewing gum, or brushing their teeth for two hours before the first session. This was checked on arrival, after which they completed training on the two rating scales and a six-question disguised mood-appetite questionnaire to later statistically control for appetitive state (described in [54]). Participants first rated hunger using a horizontal VAS followed by five descriptors presented in random order. This included fullness and thirst alongside three mood descriptors (happy, tired and anxious).

Phenotyping for sweet-liking status followed the procedure recommended by Iatridi et al. [23]. Eight 10-mL solutions were presented in clear 60 ml shot glasses at room temperature: two targets (1.0 M), four controls (two 0 M and two 0.125 M) and two unsampled dummy solutions. The target and control solutions were sampled in two blocks of three in a randomised order with a two-minute break between blocks. Following a sip and spit protocol, participants were instructed to swirl each solution around the mouth for 10 seconds, expectorate it, and rate liking and intensity before rinsing their mouth with water and tasting the next sample. After which, participants were asked to report demographic characteristics (date of birth, sex, race), current diet (i.e., omnivore, pescatarian, vegetarian, vegan) and dieting history (i.e., previous, current, or never-dieter).

##### Session 2: anthropometry

Anthropometric assessments took place in the second session following standardised instructions [55] and protocols to improve reliability as outlined in [49]. Briefly, wearing light clothing and bare feet, standing height was measured to the nearest 0.1 cm using a column scale stadiometer (SECA 220) and body composition and weight using a multi-frequency segmental bio-impedance device (MC-780MA P, TANITA). This included body weight to the nearest 0.1 kg, BF%, FFM and BMI.

#### Statistical analysis and sweet-liking phenotype screening

Consistent with previous research [23, 49, 56], participants were phenotyped based on their liking for the two 1.0 M sucrose solutions. To minimise potential confounding effects of participants not playing attention in the taste task, initial consistency checks first identified and excluded erratic responders whose liking ratings of the two 1.0 M sucrose solutions differed by >30 pt on the 100 pt scale and indicated liking (>0) for one solution and disliking (<0) for the other. Participants who rated liking for both 1.0 M sucrose solutions higher than +15 were classified as extreme sweet-likers, both below -15 as sweet-dislikers and those rating one or both 1.0 M solutions between +15 and -15 as moderate sweet-likers. Intensity gLMS, the 0.125 M control and dummy solutions were used to reduce demand effects and check that participants were engaged in the task (i.e. not rating everything as very intense).

Multiple linear regression models were run to investigate differences between phenotypes on demographic and anthropometric characteristics, and key assumptions of the general linear model were checked. The final analysis used robust linear regression models with Welch F and Games-Howell post-hoc tests to correct for unequal variance and sample size, controlling for biological sex. These are reported alongside parameter estimates and Eta squared effect sizes (*ηp*^2^): small effect <0.06; medium effect >0.06 and <0.14; large effect >0.14. For all analyses, significance was set at *p* < 0.05 and were computed using *R* Studio Version 1.4.1106 (Boston, US).

### Results

#### Sweet-liking phenotype status and demographics

Out of 286 participants who completed the phenotyping for sweet-liking status, 37 were excluded as erratic responders. A further 49 did not attend the anthropometric assessment leaving a final sample of 200 participants (extreme sweet-likers = 71; moderate sweet-likers = 93; sweet-dislikers = 36). See Table 1 for a summary of demographic and anthropometric characteristics by phenotype.

**Table 1:** Summary of demographic and anthropometric characteristics by phenotype for Experiment One and Two.

	Experiment One				Experiment Two			
	Extreme sweet-likers (*n* = 71)	Moderate sweet-likers (*n* = 93)	Sweet-dislikers (*n* = 36)	Overall (*n* = 200)	Extreme sweet-likers (*n* = 96)	Moderate sweet-likers (*n* = 153)	Sweet-dislikers (*n* = 65)	Overall (*n* = 314)
Age (years)								
mean ± s.d.	21.37 ± 2.45	21.19 ± 2.74	22.15 ± 3.34	21.43 ± 2.77	22.84 ± 3.07	24.05 ± 3.48	24.25 ± 3.65	23.72 ± 3.43
(range)	(18.5–30.1)	(18.3–32.8)	(18.1–30.9)	(18.1–32.8)	(18.9–30.6)	(18.8–33.2)	(18.8–33.7)	(18.8–33.7)
Sex, n (%)								
Male	38%, 27	35%, 33	22%, 8	34%, 68	46%, 44	42%, 64	26%, 17	40%, 125
Female	62%, 44	65%, 60	78%, 24	66%, 132	54%, 52	58%, 89	73%, 48	60%, 189
Race, n (%)								
White	76%, 54	68%, 63	67%, 24	70%, 141	69%, 66	71%, 108	63%, 41	69%, 215
Black	10%, 7	17%, 16	0%, 0	16%, 31	10%, 10	11%, 17	N/A	14%, 45
Asian	4%, 3	5%, 5	22%, 8	4%, 8	7%, 7	4%, 6	28%, 18	4%, 13
Race Not Listed	10%, 7	10%, 9	11%, 4	10%, 20	13%, 12	14%, 22	8%, 5	12%, 39
Not Recorded	N/A	N/A	N/A	N/A	1%, 1	N/A	1% 1	1%, 2
BMI								
mean±s.d.	23.2 ± 4.06	21.8 ± 2.99	22.5 ± 2.7	22.42 ± 3.4	23.7 ± 3.58	22.6 ± 3.02	22.7 ± 2.8	22.9 ± 3.19
(range)	(17–39.4)	(16–32.6)	(17.2–28.3)	(17–39.4)	(16.6–36.9)	(15.5–34.6)	(18.1–32.2)	(15.5–36.9)
Fat Free Mass								
mean±s.d.	52.4 ± 10.24	49.4 ± 9.65	47.6 ± 9.46	50.2 ± 9.95	54.8 ± 11.34	52.7 ± 10.98	49.8 ± 9.37	52.8 ± 10.89
(range)	(36.9–78.8)	(32.9–76.9)	(34.2–73.2)	(32.9–78.8)	(34.9–83.5)	(36.8– 86.8)	(35–76.3)	(34.9–86.8)
Body Fat Percentage								
mean±s.d.	21.3 ± 7.87	21.6 ± 7.74	25.2 ± 6.02	22.1 ± 7.61	21.6 ± 7.77	21.4 ± 7.23	23.5 ± 7.16	21.9 ± 7.41
(range)	(4.5–42.1)	(4.2–44.8)	(11.6–33.8)	(4.2–44.8)	(7.8–33.9)	(5.9–38.2)	(7.9–41.9)	(5.9–41.9)

Note that four participants in Experiment Two chose not to report their race.

*s.d.* standard deviation.

#### Anthropometry

Multiple linear regression models were fit controlling for sex, and a significant effect of phenotype on FFM (*F*(5, 48.35) = 46.92, *p* < 0.001, *ηp*^2^ = 0.08) and BF% (*F*(5, 49.91) = 19.61, *p* < 0.001, *ηp*^2^ = 0.05) was found but not for BMI (*F*(5, 50.53) = 1.83, *p* = 0.123, *ηp*^2^ = 0.03). However, while not significantly different, it is worth noting that on average extreme sweet-likers had a higher BMI (*M* = 23.2; *SD* = 4.06) compared to both moderate sweet-likers (*M* = 21.8; *SD* = 2.99) and sweet-dislikers (*M* = 22.9; *SD* = 2.7). Games-Howell Post Hoc tests revealed on average, extreme sweet-likers had 4.82 kg of additional FFM than sweet-dislikers (*t*(75.64) = 2.42, *p* = 0.046). For BF%, sweet-dislikers had 3.87% higher body fat compared to extreme sweet-likers (*t*(88.7) = 2.82, *p* = .016) and 3.53% higher compared to moderate sweet-likers (*t*(81.4) = 2.75, *p* = 0.02). There were no other significant differences between phenotypes for FFM and BF%.

## Experiment Two

Experiment Two aimed to replicate the findings in Experiment One in an independent sample while making two key changes to the design: firstly, by refining the protocol to determine sweet-liking phenotype status by removing the 0.125 M sucrose solution and secondly, to minimise drop-outs, both the taste and body composition sessions were scheduled on the same day.

### Participants and methods

#### Participants, procedures and statistical analysis

Experiment Two recruited 338 different adults, aged 18–34, from the University of Sussex and the local area (Brighton, UK) to take part in a two-session lab-based study also advertised as ‘Taste, Personality and Body Composition’ under the same ethical approval. The study was designed to replicate the findings in Experiment One and followed the same procedures (including informed consent and debriefing), phenotyping and analysis but with a refined protocol to screen for sweet-liking phenotype status using only two 1.0 M solutions (342.3 g g/vol) and two water controls (0 M), excluding the 0.125 M samples. This meant only four 10-mL solutions were sampled from the eight presented: two targets (1.0 M) and two controls (0 M) with four unsampled dummy solutions. These were sampled in two blocks of two, first 0 M and then 1.0 M, with a two-minute break between blocks. In addition, around 20% of our participants did not attend their anthropometric assessment in Experiment One. Therefore, participants participated in both sessions on the same day, with their anthropometric assessment in the morning (08:00-11:00) and the phenotyping for sweet-liking status two hours after eating breakfast (10:30–14:00).

### Results

#### Sweet-liking phenotype status and demographics

Of the 338 participants who completed both sessions, 22 were excluded as erratic responders (see Experiment One for post-participation exclusion criteria), and a further 2 who provided contradictory medical information at pre-screening, leaving a final sample of 314 participants (extreme sweet-likers = 96; moderate sweet-likers = 153; sweet-dislikers = 65). See Table 1 for a summary of demographic and anthropometric characteristics by phenotype.

#### Anthropometry

Multiple linear regression models were fit controlling for sex, and a significant effect of phenotype on BMI (*F*(5, 99.33) = 4.88, *p* < 0.001, *ηp*^2^ = 0.03), FFM (*F*(5, 96.69) = 113.95, *p* < 0.001, *ηp*^2^ = 0.07) and BF% (*F*(5, 101.59) = 39.21, *p* < 0.001, *ηp*^2^ = 0.02) was found but it appeared the significant effect of BF% was driven by sex effects. Although not significantly different, as in Experiment One, it is worth noting that on average sweet-dislikers still had the highest BF% (*M* = 23.54; *SD* = 7.16) compared to both extreme sweet-likers (*M* = 21.61; *SD* = 7.77) and moderate sweet-likers (*M* = 21.36; *SD* = 7.23). Differences in ages between samples may explain this effect not replicating (*t*(484.4) = –8.32, *p* < 0.001), with participants in Experiment Two (*M* = 23.72, *SD* = 3.43) being significantly older than in Experiment One (*M* = 21.43, *SD* = 2.77). Games-Howell Post Hoc tests revelled, on average, extreme sweet-likers had 1.11 kg/m2 higher BMI than moderate sweet-likers (*t*(177.06) = 2.53, *p* = 0.033). For FFM, extreme sweet-likers had 4.98 kg of additional FFM than sweet-dislikers (*t*(152.7) = 3.04, *p* = 0.008). There were no other significant differences between phenotypes for FFM and BMI.

## Mid discussion

In the first part of this paper, we introduced two previously unpublished datasets that investigated the impact of sweet-liking phenotype classification on three anthropometric measures: BMI, BF% and FFM. In Experiment One, contrary to the idea that sweet liking drives obesity, sweet-dislikers had a significantly higher BF% compared to extreme sweet-likers and moderate sweet-likers. Although not significant, Experiment Two displayed a similar trend. In our previous multi-country study, participants were median split by age, revealing that being an extreme sweet-liker was associated with lower body fat in the younger and not the relatively older (>21 years) group [49]. We speculated that age groups reflected different levels of exposure to the obesogenic environment. Here, participants in Experiment Two were significantly older than those in Experiment One (Table One). Furthermore, both studies revealed a significant effect of phenotype on FFM, with extreme sweet-likers displaying higher FFM than sweet-dislikers. Although not significantly different from sweet-dislikers, extreme sweet-likers also had, overall, the highest BMI in both studies.

Taken with the limited literature using the three-phenotype model, this supports our previous suggestion that the higher BMI often observed in sweet-likers may be attributed to a greater FFM [49]. However, as these data are only from one research group in a young, predominantly westernised population, and the results did not replicate perfectly across both studies we presented here, further investigations to elucidate differences are needed.

Therefore, to address this and broader issues in the literature in finding a consensus on whether sweet liking is a key driver of obesity (i.e., relatively small samples, an overreliance on BMI and a lack of phenotyping), we conducted a systematic literature review and employed an IPD meta-analysis. Specifically, this approach provides more precise and less biased estimates of effect and can better account for parameter correlations [57]. It also enables us to apply standardised phenotyping across datasets and maximise power to detect true effects [58], including exploring potential moderators of the effect of sweet-liking phenotypes on the anthropometric measures. This includes factors of study variability (e.g., sucrose concentration, repetition, and place of data collection) and participant characteristics (e.g., biological sex, age, and race). In the final section of this paper, we present the results of multiple IPD meta-analyses to elucidate differences between the sweet-liking phenotypes and four anthropometric measures of interest: BMI, BF%, FFM, and waist circumference (WC).

## Systematic review and IPD meta-analyses

### Methods

#### Eligibility criteria

All primary studies published in English on non-clinical adult human participants or control participants in clinical studies (18+ years) were considered from 1st January 1970 to 19th July 2021. This period was selected to coincide with the year of the first publication by Pangborn [28], identifying the sweet-liking phenotypes. A second follow-up search was conducted during analysis and no further papers were added. To be eligible for inclusion, studies had to include a taste assessment that measured liking using aqueous sucrose solutions (above 5 ml and 13.7% w/v or 0.4 M) on a scale which allowed calculation of the three sweet-liking phenotypes and an anthropometric measure of interest: BMI, BF%, FFM or WC. The taste assessment must have measured liking specifically and not only preference, for example, not only a forced-choice paired-comparison task, as participants can prefer a solution without liking it [50]. Additionally, the liking scales had to be precise enough to discriminate the three phenotypes (i.e., generalised labelled magnitude scales [gLMS], visual analogue scales [VAS] and 9-point Likert scales) [59]. We chose only to include liking for sucrose solutions and not food samples, as when sucrose is manipulated in food products, the food matrix and prior memory can influence liking beyond sweet taste. Solutions had to be a minimum of 5 ml to allow the solution to be sufficiently swilled around the mouth and above 0.4 M sucrose in strength. This is the lowest concentration at which the three phenotypes can be readily discriminated, with the clearest distinction at 1.0 M sucrose [23].

#### Study identification and selection process

The analysis complied with the Preferred Reporting Items for Systematic Review and Meta-analyses (PRISMA) IPD Statement [60]. The search protocol was designed by R.A. and agreed with M.Y. Four databases were searched: Web of Science, Scopus, PubMed and PsycINFO, using combinations of the key research concepts: (“sucrose*”) AND (“sweet taste*” OR “sweet liking phenotype*” OR “sweet liking*” OR “sweet liker*” OR “sweet preference*” OR “taste preference*” OR “pleasantness*” OR “hedonics*”). Anthropometric measures, particularly BMI, are often included in demographics sections but are not necessarily a core aim of the research paper and, hence, are not reflected in the abstract or title. Therefore, key search terms relating to anthropometric measures were left out, and eligible full-text articles were screened manually. R.A. extracted all records from the four databases into EndNote. EndNote automatically removed duplicates before R.A. manually checked for any it had missed. Titles and abstracts were then independently screened by R.A. and M.Y. before assessing full-text articles. The researchers resolved disagreements through discussion and joint assessment of the full-text articles. When requesting the data, researchers also asked if the authors had or were aware of any unpublished data that met our criteria.

#### Data collection and data items

Authors for all eligible studies were contacted by email three times (September 2021, December 2021, and February 2022) with an outline of the proposed project, confirmation of study details (i.e., sucrose strength, volume and anthropometric measurements) and a request for demographic and anthropometric individual participant data. Studies were excluded from the analyses if no response was received after three attempts. Authors were asked to provide participant-level variables: demographic information (sex, age, and race), anthropometric data (BMI, BF%, FFM, or WC), liking and intensity ratings of any sucrose solutions above 0.4 M and study-level variables (sucrose concentration(s) used, number of sucrose repetitions and country of data collection).

Sweet-liking data were collated, cleaned and phenotyped by R.A. using Excel and checked by M.Y. First, liking ratings had to be standardised by transforming all VAS and gLMS scales into 100-point scales (–50 to +50). Next, they were phenotyped using the statistically determined criteria applied in Experiments One and Two [23, 56]. Where two sucrose solutions were rated, those with a difference of >30 between scores that crossed 0 (i.e., a rating of –15 and +20) were labelled erratic responders and excluded from analysis. For remaining participants, liking ratings for both sucrose solutions had to be higher than +15 for classification as an extreme sweet-liker, below -15 for a sweet-disliker, and everyone else was classified as a moderate sweet-liker. For studies whereby one sucrose solution was rated, liking had to be higher than +15 for classification as an extreme sweet-liker, below –15 for a sweet-disliker or between –15 and +15 for a moderate sweet-liker. However, as this phenotyping criterion was created based on two 1.0 M sucrose solutions, which did not match the protocols of all the studies, participants who rated solutions weaker than 1.0 M were re-phenotyped using adjusted cut-offs from a sensitivity specificity analysis [23]. In addition, for studies using a 9-point Likert scale, participants were phenotyped similarly with three cut-offs: 0–3 sweet-dislikers, 4-6 moderate sweet-likers, and 7–9 for extreme sweet-likers.

#### Risk of bias assessment in individual studies

To our knowledge, there are no standardised guidelines for assessing data quality in non-medical human experimental data collected without interventions or randomisation (i.e., not using randomised control trials, cohort or case-control studies). Therefore, like Albers et al. [61] we have adapted the study quality assessment tool from National Institute of Health and excluded questions not applicable to the research we have included (e.g., selection bias, randomisation methods, intervention integrity) and discuss the aspects of data quality relevant to the screening criteria and studies included (e.g., recruitment and data collection method, classification method, study and participant level characteristics included).

#### Statistical analysis: IPD meta-analysis

All analyses were performed in *R* version 4.2.2 (2022), using the packages lme4 [62] and robustlmm [63] for fitting multi-level models and the tidyverse package suite [64] for data wrangling. Data analysis was completed by R.A. and M.S. Multiple one-stage IPD meta-analyses were run using Iatridi and colleagues’ phenotyping criterion and the associated sensitivity specificity analysis for phenotype classification [23]. A separate analysis was conducted for each anthropometric measure, with participants nested within studies first checking assumptions of the general linear model. To ensure extreme cases did not drive effects, we ran a sensitivity analysis with robust models using the DAS-tau adaptation of the *M-*estimator [63, 65] and models excluding participants with BMI > 40.

For the primary analyses, exploring the effects of sweet-liking phenotypes on each of the anthropometric measures, we built the multi-level linear models sequentially comparing model fit at each stage: first assessing the random intercept model to judge if the model fit has improved by allowing the intercepts to vary, then adding fixed effects of phenotypes and biological sex.

The models were fitted using maximum likelihood estimation and took the following form:

$$\begin{array}{ll}{\hat{\text{anthropometric measure}}}_{{ij}}={\hat{b}}_{0}+{\hat{b}}_{1}{\text{Phenotype}(\text{moderate sweet}-\text{likers vs extreme sweet}-\text{likers})}_{{ij}}\\ \qquad\qquad\qquad\qquad\qquad\qquad\quad+\,{\hat{b}}_{2}{\text{Phenotype}(\text{sweet}-\text{dislikers vs extreme sweet}-\text{likers})}_{{ij}}\\ \qquad\qquad\qquad\qquad\qquad\qquad\quad+\,{\hat{b}}_{3}{\text{Sex}(\text{female vs male})}_{{ij}}\\ \qquad\qquad\qquad\qquad\qquad\qquad\quad+\,{\hat{u}}_{0j}+{\hat{u}}_{1j}+{e}_{{ij}}\end{array}$$

wherein the anthropometric measure (BMI, FFM, BF% or WC) is predicted from the fixed effects of phenotype and sex. For phenotype, extreme sweet-likers were coded as the baseline category against which moderate sweet-likers and sweet-dislikers were compared. ${\hat{b}}_{1}$ and ${\hat{b}}_{1}$ thus represent the difference on a given outcome between each respective phenotype and the baseline. Sex was included in the models as a covariate, with males being set as the baseline category. We calculated Cohen’s *d* for each contrast to allow comparison of effects across predictors and outcomes: small <0.2, medium <0.5 and large effects <0.8 [66, 67]. *p* values for the contrast were estimated using Satterthwaite’s method [68].

We then tested whether available participant-level demographic information or study-level characteristics moderated the effect of the sweet-liking phenotypes on our anthropometric measures. Each potential moderator was included in a separate multi-level mixed-effects linear regression model to examine the main effects and interactions with the sweet-liking phenotypes on our anthropometric measures.

### Results

#### Systematic search

**Fig. 2:**
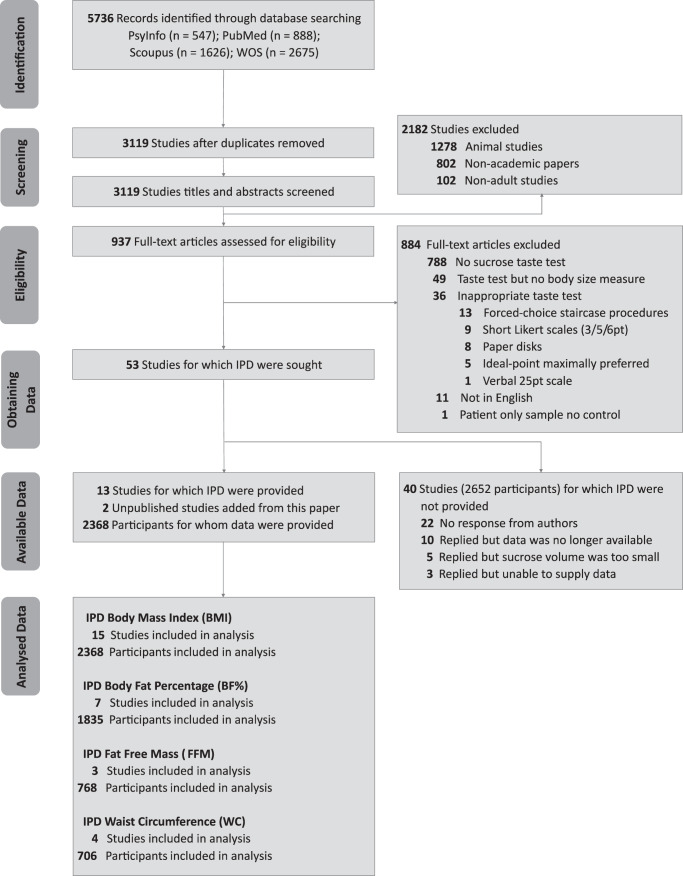
Individual participant data PRISMA flow diagram. The IPD PRISMA flow diagram indicating the number of studies retained and excluded at each stage of the review process.

Our search identified 5736 potential articles, including 2617 duplicates. The titles and abstracts of the remaining 3119 record abstracts and titles were screened, with 937 meeting our criteria for full-text screening. In total, 53 studies met our search criteria. Of those, 13 datasets were received [22, 26, 42, 49, 69–77] and combined with the two presented in this paper. This is summarised in Fig. 2, the PRISMA flow diagram.

#### Study and participant characteristics

The final sample included 15 studies (*n* = 2368: 1413 females and 955 males), as summarised in Table 2. All studies collected BMI (*n* = 2368), seven BF% (*n* = 1835), three FFM (*n* = 768) and four WC data (*n* = 706). Studies were conducted in different geographic regions, including Israel, Japan, Malaysia, Poland, Singapore, UK and USA; however, three studies did not record the ethnicity or race of individual participants, and one did not provide individual ages, although all participants were under 35. Table 3 presents participant characteristics by anthropometric measure and sweet liking phenotype categorised using Iatridi et al.’s criterion [23]. To confirm phenotype characterisation for studies that had solutions weaker than 1.0 M, cut-offs from a sensitivity specificity analysis were used to re-phenotype participants. Specifically, six of the 15 studies need re-phenotyping [22, 42, 70, 73, 75, 76]. This adjusted the sweet-disliker cut-offs from –15 to –5 [42, 70, 73] or 0 [75]. Two studies did not provide raw liking data [22, 76], so could not be re-phenotyped. However, these were already based on hierarchal cluster analysis that Iatridi’s criterion employs.

**Table 2:** Characteristics of the studies included in the current systematic review and independent participant data meta-analysis.

Studies	Study-level variables					Participant-level variables								
	Sucrose Molarity	Solution repetitions	Liking assessment method	Original Rating range	Country of collection	Age range (*M)*	Race of participants	Biological sex		Anthropometric Outcome				
								Male	Female	Total *n*	BMI	BF%	FFM	WC
Experiment One	0.83 M	2	VAS	±50	England	18–32 (21)	Asian, Caucasian, Black & Other	68	132	200	✓	✓	✓	
Experiment Two	1.0 M	2	VAS	±50	England	18–33 (24)	Asian, Caucasian, Black & Other	125	189	314	✓	✓	✓	
Bouhlal et al. [69]	0.83 M	5	VAS	0-200	USA	22–58 (43)	Asian, Caucasian, Black & Other	44	12	56	✓			
Dubovski et al. [70]	0.5 M	1	9 pt scale	9 pt	Israel	---	Caucasian	4	14	18	✓			
Garneau et al. [22]	0.4 M	1	VAS	0-100	USA	18–92 (42)	Asian, Caucasian, Black & Other	244	403	647	✓	✓		
Hartman-Petrycka et al. [71]	0.88 M	3	VAS	±5	Poland	30–77 (56)	Caucasian	105	60	165	✓			
Hayes & Duffy [73]	0.58 M	2	gLMS	±100	USA	19–39 (26)	---	43	37	80	✓			✓
Hayes et al. [72]	0.96 M	3	gLMS	±100	USA	21–65 (32)	---	28	24	52	✓			✓
Iatridi et al. [49]	1.0 M	2	VAS	±50	USA/ England	18–35 (23)	Asian, Caucasian, Black & Other	79	175	254	✓	✓	✓	✓
Lim et al. [26]	1.0 M	2	VAS	±50	Singapore	21–50 (30)	Asian	0	66	66	✓	✓		
Methven et al. [74]	1.05 M	1	VAS	0-100	England	18–50 (29)	Asian and Caucasian	12	23	35	✓			
Nishihara et al. [75]	0.7 M	10	gLMS	±100	Japan	21–64 (48)	Asian	0	51	51	✓			
Szczygiel et al. [76]	0.7 M	1	VAS	0-100	USA	18–37 (24)	---	14	26	40	✓	✓		
Thai et al. [42]	0.55 M	1	VAS	0-10	Malaysia	18–77 (29)	Asian (Indian, Chinese, Malayian)	159	166	325	✓	✓		✓
Yeomans et al. [77]	0.83 M	2	VAS	0-100	England	18–41 (23)	---	19	38	57	✓			

Note that one study did not record ages of participants and four studies did not collect data on the race of participants.

*BF%* body fat percentage, *BMI* body mass index, *FFM* fat free mass, *gMLS* generalised labelled magnitude scale, *M* mean; *VAS* visual analogue scale, *WC* waist circumference.

**Table 3:** Characteristics of the participants included in the current systematic review and independent participant data meta-analysis split by anthropometric outcome and phenotype.

Anthropometric outcome																
	Body mass index				Body fat percentage				Fat free mass				Waist circumference			
Participants by phenotype, n (%)	Extreme sweet-likers	Moderate sweet-likers	Sweet-dislikers	Overall	Extreme sweet-likers	Moderate sweet-likers	Sweet-dislikers	Overall	Extreme sweet-likers	Moderate sweet-likers	Sweet-dislikers	Overall	Extreme sweet-likers	Moderate sweet-likers	Sweet-dislikers	Overall
	682 (29%)	1063 (45%)	623 (26%)	2368	527 (29%)	814 (45%)	494 (36%)	1835	237 (31%)	374 (49%)	157 (20%)	768	120 (17%)	3289 (41%)	297 (42%)	706
Anthropometric outcome																
Mean ± s.d.	25.47 ± 5.48	24.75 ± 5.25	24.73 ± 5.58	24.95 ± 5.41	26.1 ± 10.08	25.7 ± 9.9	25.98 ± 10.34	25.89 ± 10.05	53.3 ± 11.09	51.1 ± 10.49	48.2 ± 8.94	51.2 ± 10.53	80.99 ± 12.51	80.98 ± 13.44	82.57 ± 14.36	81.7 ± 13.69
Range	16.1–57.1	14.5–55.8	15.2–63.6	14.5–63.6	2.5–57.1	1.4–55.7	3.3–71.5	1.4–71.5	34.9–84.3	32.9–86.8	34.2–76.3	32.9–86.8	58.3–122.4	59.7–137.2	58.4–149.9	58.3–149.9
Age (years)																
Mean ± s.d.	33.24 ± 15.77	32.93 ± 15.3	32.79 ± 15.26	32.98 ± 15.42	30.25 ± 12.01	31.79 ± 15.29	29.21 ± 13.2	30.65 ± 14.42	22.1 ± 3.15	22.9 ± 3.7	23.1 ± 3.75	22.7 ± 3.57	23.97 ± 7.89	25.45 ± 8.57	28.7 ± 14.01	26.57 ± 11.26
Range	18–80	18–92	18–79	18-92	18-80	18-92	18-79	18-92	18–34	18–35	18–34	18–35	18–59	18–66	18–77	18–77
Sex, n (%)																
Male	297 (44%)	425 (40%)	233 (37%)	955 (40%)	207 (39%)	313 (38%)	166 (34%)	686 (37%)	96 (41%)	141 (38%)	35 (22%)	272 (35%)	50 (42%)	133 (46%)	124 (42%)	307 (44%)
Female	385 (56%)	638 (60%)	390 (63%)	1413 (60%)	320 (61%)	501 (62%)	328 (66%)	1149 (63%)	141 (59%)	233 (62%)	122 (78%)	496 (65%)	70 (58%)	156 (54%)	173 (58%)	399 (56%)
Race, n (%)																
Caucasian	444 (65%)	628 (59%)	234 (38%)	1306 (55%)	348 (66%)	574 (71%)	147 (30%)	1069 (58%)	177 (75%)	274 (73%)	104 (66%)	555 (72%)	57 (48%)	103 (36%)	38 (13%)	198 (28%)
Asian	86 (13%)	185 (18%)	330 (52%)	601 (25%)	78 (15%)	137 (17%)	314 (63%)	529 (29%)	21 (9%)	44 (12%)	37 (24%)	103 (13%)	35 (29%)	66 (23%)	246 (83%)	347 (49%)
Black	26 (4%)	33 (3%)	10 (2%)	69 (3%)	18 (3%)	18 (2%)	N/A	36 (2%)	14 (6%)	12 (3%)	N/A	26 (4%)	4 (3%)	1 ( < 1%)	N/A	5 (<1%)
Race Not Listed	54 (8%)	77 (7%)	24 (4%)	155 (7%)	54 (10%)	76 (9%)	23 (5%)	153 (8%)	24 (10%)	44 (12%)	16 (10%)	84 (11%)	5 (4%)	13 (4%)	7 (2%)	25 (4%)
Not Recorded	72 (11%)	140 (13%)	25 (4%)	237 (10%)	29 (6%)	9 (1%)	10 (2%)	48 (3%)	N/A	N/A	N/A	N/A	19 (16%)	106 (37%)	6 (2%)	131 (19%)

Note that one study did not record ages of participants and four studies did not collect data on the race of participants.

*s.d.* standard deviation.

#### Risk of bias assessment

Due to strict screening criteria in selecting studies for inclusion (see IPD meta-analysis eligibility criteria), the data quality across studies relevant to the protocols assessed here appeared to be of a similar high quality (Table S1, S2 in supplementary materials).

#### One-stage IPD meta-analysis

Separate mixed-effects linear models were fit for each anthropometric measure, whereby adding sex and phenotype improved model fit. For all outcomes, there was a small variation in intercepts across the studies (*SD*_*BMI*_ = 2.3, *SD*_*FFM*_ = 1.1, *SD*_*BF%*_ = 2.9, *SD*_*WC*_ = 4.37). This variation reduced when fixed effects were added into the model (Table 4).

**Table 4:** Summary of fixed and random effects across anthropometric outcome and phenotype while holding sex constant.

	Body mass index					Fat free mass					Body fat percentage					Waist circumference					
	Random intercepts		+ Fixed effects			Random intercepts		+ Fixed effects			Random intercepts		+ Fixed effects			Random intercepts		+ Fixed effects			
Fixed effects	*b*	*SE*	*b*	*SE*	*p*	*b*	*SE*	*b*	*SE*	*p*	*b*	*SE*	*b*	*SE*	*p*	*b*	*SE*	*b*		*SE*	*p*
Intercept	24.47	0.61	25.41	0.63	<0.001	51.01	0.74	63.7	0.59	<0.001	24.76	1.14	18.85	1.16	<0.001	83.07	2.28	88.44		2.38	<0.001
Phenotype (MSL vs ESL)			–0.69	0.24	0.006			–1.75	0.51	<0.001			–0.82	0.48	0.085			–1.13		1.38	0.412
Phenotype (SD vs ESL)			0.14	0.30	0.651			–1.96	0.64	0.002			0.52	0.60	0.380			0.81		1.48	0.583
Sex (female vs male)			–1.07	0.21	<0.001			–17.54	0.47	<0.001			8.87	0.42	<0.001			–9.29		0.97	<0.001
Random effects																					
Level 2 (study ID)	2.3		2.22			1.1		0.59			2.9		2.76			4.37			3.93		
Level 1 (residual)	4.94		4.91			10.46		6.12			9.48		8.45			13.25			12.47		
Deviance	14334.3		14299.97			5789.1		4966.74			13489.2		13067.6			5665			5577.7		

*ESL* extreme sweet-liker, *MSL* moderate sweet-liker, *b* parameter estimates, *p p* value, *SE* standard error, *SD* sweet disliker.

**Fig. 3:**
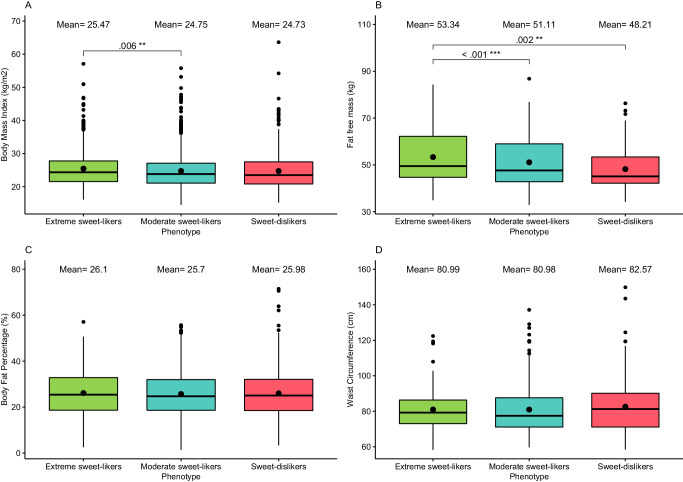
A comparison of the four key anthropometric outcomes across the three sweet-liking phenotypes adjusted for sex. A comparison of BMI (**A**), FFM (**B**), BF% (**C**) and WC (**D**) across the three sweet-liking phenotypes adjusted for sex. Boxes represent the interquartile ranges, whiskers the minimum and maximum score of each anthropometric outcome, and solid lines the medians. The means for each phenotype are presented at the top of each relevant column. Significant differences estimated using Satterthwaite’s method are denoted with an asterisk (*p < 0.05, **p < 0.01, ***p < 0.001).

No statistically significant differences in WC or BF% were found between the three phenotypes while holding sex constant. For BMI, moderate sweet-likers showed significantly lower BMI compared to extreme sweet-likers, although this effect was small (*b* = –0.69, 95% CI (–1.17, –0.20), *d* = –0.11, *p* = .006). Sweet-dislikers’ BMI was, on average, lower than that of extreme sweet-likers (*b* = 0.14), however, this difference was not statistically significant (*p* = .651). Most interestingly for FFM, both sweet-dislikers and moderate sweet-likers showed significantly lower FFM than extreme sweet-likers (*b* = –1.96, 95% CI (–3.21, –0.71), *d* = –0.22, *p* = 0.002; *b* = –1.75, 95% CI (–2.75, –0.75), *d* = –0.25, *p* < 0.001) respectively. See Fig. 3. This pattern of results was consistent with models using robust estimation and excluding participants with a BMI > 40 and replicated when participants were re-phenotyped using the adjusted cut-offs from the sensitivity specificity analysis (Table S3 in supplementary materials).

#### Moderators and interaction effects

Participant-level moderators of sex, race and age improved model fit with significant main effects across each anthropometric measure (Table S4–S6 in supplementary materials). However, only four significant interaction effects were found after applying robust estimation. Specifically, sex moderated the relationship between phenotype and BF% (*t*(1831.77) = –3.99, *p* < 0.001) and WC (*t*(701.57) = –2.59, *p* = 0.01), where compared to male extreme sweet-likers, female sweet-dislikers had 4.42% higher body fat and female moderate sweet-likers had 7 cm wider WC. In addition, on average younger extreme sweet-likers had the smallest WC but as age increased, WC increased at a quicker rate in extreme sweet-likers compared to sweet-dislikers (*t*(703.8) = –3.59, *b* = *–*0.53*, p* < 0.001) and moderate sweet-likers (*t*(705.81) = –2.1, *b* = *–*0.34*, p* = 0.03).

There were no fixed or interaction effects for the study level moderators of sucrose concentration or number of repetitions of sucrose solutions across any of the anthropometric measure (Table S7, S8 in supplementary materials). However, country and continent of data collection significantly improved model fit with significant main effects for BMI, FFM and WC but not BF% (Table S9, S10 in supplementary materials). There were no interaction effects for any of the study level moderators.

## Discussion

This paper investigated differences between sweet-liking phenotypes on four anthropometric measures: BMI, BF%, FFM, and WC and aimed to identify potential moderators. Our findings revealed significant main effects of phenotype on BMI and FFM and interaction effects for BF% and WC. For BMI, our findings align with the general view accepted in the popular media that a higher BMI is associated with an increased liking for sweet tastes e.g., [29–33], although in most studies this trend does reach significance [50, 78]: we found that extreme sweet-likers had the highest BMI, although this was only statistically significantly different from moderate sweet-likers. However, it is important to recognise that high sugar intake is considered more detrimental than beneficial for health [34] and here we are discussing the link between high liking for sweet tastes and an unhealthier body size, where the evidence supporting this widespread belief is more mixed [50]. Most interestingly, however, the higher BMI often attributed to people with increased liking for sweet tastes may be due to larger FFM not body fat: here, extreme sweet-likers had significantly greater FFM than sweet-dislikers and moderate sweet-likers.

Overall, these findings support those of Iatridi et al.’s [49] and align with well-established evidence indicating a positive association between FFM and energy requirements [79, 80], where efferent signals from the body’s energy reserves (e.g., adipose tissue) and metabolically demanding tissues and organs (e.g., FFM) integrate centrally with afferent signals from the sensory properties of taste stimuli contributing to the final hedonic response e.g., [81, 82]. This reinforces the long-standing idea that the expression of sweet liking may partly reflect homoeostatic energy needs e.g., [43, 83–85]. However, caution must be taken when generalising these findings as the FFM data is from the same research group in young (18–35 years) westernised populations (UK and USA).

We consider two discrete but complementary frameworks to interpret the relationship between sweet-liking and body composition: alliesthesia and interoception. Alliesthesia is a psychophysiological phenomenon whereby the usefulness of the stimulus for the internal body determines the experienced pleasure [83, 86]. Specifically for sweetness, shifts in liking are modulated by motivational state: hunger enhancing perceived pleasantness and satiation reducing liking e.g., [25, 87]. These shifts have been observed in naturally occurring increased need states, such as periods of rapid development where sweetness liking is heightened e.g., [88, 89] and in pharmacologically induced need states resulting in increased liking during hypoglycaemia or decreased liking after glucose loads [86]. Alliesthesia, is often discussed alongside sensory-specific satiety: a decrease in the associated pleasantness of the sensory characteristics of eaten food (i.e., taste, texture, shape, temperature, colour) relative to foods that have different sensory characteristics that have not been eaten [90]. Here, sensory-specific satiety would suggest that eating sweet-tasting products would reduce the attractiveness of other sweet-tasting products [91]. Critically, in alliesthesia a decrease in liking of sweet-tasting products is expected through the metabolic effects of sugar after ingestion [92], whereas sensory-specific satiety it is the sensory stimulation experienced when ingesting the sweet food that decreases liking, not the metabolic effects [90]. Although some suggest that sensory-specific satiety is just a form of negative alliesthesia [93].

Complementary to alliesthesia is interoception, defined as the ability to perceive bodily sensations from internal systems, including homoeostatic and emotional needs [94]. Interestingly, preliminary evidence suggests that extreme sweet-likers have higher internal awareness of their bodies and interoceptive appetite cues [56]. Specifically, compared to sweet-dislikers, they perform better in sensing generic interoceptive signals (heartbeats), signals associated with gut-brain communication (gastric satiation and fullness), and display behavioural eating patterns characterised by a reliance on internal signals (i.e., mindfulness and intuitive eating, trait hunger and intensity seeking) [56]. Notably, the exposure to the obesogenic environment and subsequent overconsumtpion of hyperpalatable foods has been posited to interfere with the ability to regulate food intake [95] and process the rewarding properties of foods [96]. Therefore, in modern affluent food environments, efficient interoceptive mechanisms, i.e., being attuned to internal body signals, might be essential if hedonic responses to sweetness (i.e., explicit liking) represent the internal need state of the body (i.e., implicit wanting) with alliesthesia serving as the foundation for the interplay between the body’s homoeostatic systems and sweet-liking.

The narrative above seems to find some support in our findings. Firstly, like Iatridi et al.. [40], age significantly moderated the relationship between phenotype and WC: on average younger extreme sweet-likers had the lowest WC but the highest measurement as age increased, and they likely experienced increased effects of the obesogenic environment. Secondly, beyond the expected and well-established effects of biological sex and age on all measures of body composition [97], here, significant moderation effects of sex between phenotype and BF% as well as WC were revealed. Specifically, male extreme sweet-likers exhibited lower BF% and WC compared to male sweet-dislikers, while female extreme sweet-likers had the highest BF% and WC compared to female sweet-dislikers. To interpret these, we must consider the broader implications of sex on body composition, where males biologically possess a lower BF% and higher FFM than females [97]. Therefore, if FFM is the anthropometric measure most likely to contribute to the expression of taste hedonics, it is reasonable to anticipate that the influence of FFM would be more pronounced in males. However, for the young females in this study, who are relatively non-overweight, differences in BF% were within a narrow range; therefore, downregulating signals from the adipose tissue could have been less potent and, consequently, amplified the impact of interoceptive sensitivity on their hedonic responses to sweetness, potentially masking any protective signals from lower FFM.

Indeed, compared to sweet-dislikers, female extreme sweet-likers reported increased food intake in response to negative emotions [56] and increased positive emotions to sweet foods [98], suggesting greater responsiveness to internal cues. Here, emotional responses to sweet foods appeared to be influenced more by product liking in sweet-dislikers and sweetness levels for extreme sweet-likers [98]. Elsewhere, extreme sweet-likers have shown heightened sensitivity to reward [49], a trait associated with eating for pleasure. Although no direct effect of phenotype on BF% or WC was found when controlling for sex, it could be hypothesised that female extreme sweet-likers, less exposed to the beneficial effects of FFM, may make ingestive decisions driven by their emotional state, leading to overconsumption of foods that stimulate reward circuits, ultimately resulting in relatively higher BF% and WC than their sweet-disliker counterparts. Interestingly, modern humans are considered less efficient in recruiting homoeostatic mechanisms that resist upward deviations from their weight set point [99]. This may further explain the diminished anorexigenic effects of female extreme sweet-likers’ larger adipose tissue.

While it is crucial to emphasise that the reasons for these complex interactions between sweet-liking phenotype, anthropometric measures, and eating behaviour remain uncertain and require further investigation, it could be proposed that this relationship is likely to be underpinned primarily by the relative potency of homoeostatic signals (e.g., from FFM), and secondarily, by one’s sensitivity to broader interoceptive signals which, depending on the level of exposure to the obesogenic environment, may determine whether other traits, such as being emotional eaters will repeatedly fail to balance their energy intake with their metabolic requirements.

While alliesthesia and interoception offer one potential explanation, we also acknowledge that many wider factors will contribute to our liking for sweet tastes [100], our overall food choices [101] and eating decisions e.g., [52, 102, 103] which may further interrelate and act as protective or risk factors in influencing our current and future food selection and eating outside our nutritional needs. While excess energy is the fundamental mechanism for developing overweight and obesity, a set of multi-factorial aetiologies have been identified: from human biology to behaviour, including our physical and social environment, that affect the balance between the energy stored and the energy the body uses [104]. Our findings here about sweet-liking-anthropometry associations could be moderated by one or more of these factors. Previously, we have reported significant differences between extreme sweet-likers and sweet-dislikers in behavioural traits such as reward sensitivity and sensation seeking but not in relation to restraint eating or disinhibition [49]. In that study, the younger and older extreme sweet-likers were presented with the lowest BF% and highest FFM, respectively. Additionally, it was hypothesised that exposure to the obesogenic environment may impact the direction of the effect of sweet-liking on body composition [49]. Therefore, cognitive processes that relate to the consequences of consuming sweet-tasting foods and, hence, likely to prevent or decrease intake, may explain some additional variance in the sweet-liking-obesity relationship [105, 106].

Prior memory and learning about the metabolic and hedonic consequences of consuming a specific food are also known mediators of food choice [82] and may place sweetness and sugars with their self-reinforcing properties e.g., [20, 21] more prone to overconsumption. A recent 2023 neuroimaging study confirmed that repeated exposure to highly palatable diets causes enhanced brain response to sweet and fatty foods, with the neurobehavioral adaptations observed supporting adaptive associative learning [96]. Likewise, repeated exposure to highly palatable foods was recently reported to weaken appetite control in humans, an effect that was partly explained by a decline in hippocampal-dependent learning and memory performance [95]. However, both the link between sweet-liking phenotypes and dietary intake [50] and the ‘addiction-like’ effects of sweet taste exposure on intake have been largely inconclusive e.g., [107–109]. However, it is worthwhile noting that limited, well-powered studies adequately characterise exposures and control for known covariates over a sufficient period, although promising randomised control trials that directly address these issues are currently underway e.g., [110–112].

### Strengths, limitations and future directions

One of the biggest strengths of this research is its robust methodology. Specifically, employing one-stage IPD meta-analyses yielded more precise and less biased estimates of effect, accounted for parameter correlations [57] and allowed us to standardise phenotyping criteria while exploring potential moderators across a range of anthropometric measures not possible in single experimental studies [58]. However, we encountered issues accessing relevant data and could only include 32.5% of known studies. Reasons included the data no longer being available, data holders retiring, and data legislation precluding sharing (e.g., data not allowed to leave the EU: the current analysis was run in the UK after Brexit). In addition, despite our efforts to locate relevant unpublished and unregistered datasets, like Experiments One and Two presented in this paper, there are likely relevant data of which we are unaware. This highlights the need for greater integration of open science practices into research protocols, including pre-registering studies and sharing analysis scripts and datasets to support future meta-analyses and tackle broader research issues, including publication bias and the replication crisis [113, 114].

Although this meta-analysis considered different anthropometric measures to overcome the limitations of BMI in differentiating between the body’s tissues and tissue distribution [48], studies reporting body composition data primarily used bioimpedance analysis and were predominantly from affluent countries. Therefore, anthropometric results must be interpreted cautiously, although we note that key findings remained consistent when controlling for race. The lack of studies in more deprived regions may reflect barriers such as the relative high costs and complexities associated with body-composition analyses [115, 116]. Future studies examining these relationships in more diverse and less westernised populations (i.e., greater range of age, cultures, socioeconomic and health status) will be critical to draw more generalised conclusions and disentangle the multifaceted obesity aetiology. In addition, more longitudinal research is needed to investigate how anthropometric profiles, eating habits, and taste hedonic patterns vary over time, including in cultures with lower obesity rates and/or lower exposure to hyperpalatable foods and diets to further elucidate potential protective factors [50, 78].

### Conclusion

To conclude, by using adiposity measures beyond BMI, in this research, we were able to challenge the widely accepted belief that those who most like sweet taste (i.e., extreme sweet-likers) are likely to present with excess adiposity and while they may hold a higher BMI in many studies, this is likely driven by an increased FFM. Thus, expression of sweet liking may partly reflect metabolic need and be modulated by need state, potentially explaining enhanced interoceptive abilities in those who most like sweet taste. In summary, this work questions the simple model where sweet liking alone is a risk factor for obesity and ultimately an independent contributor to obesity’s disease burden, but this still leaves many questions unanswered, like what combination of behavioural, biological and environmental factors may influence the observed anthropometric variation by sweet-liking phenotype.

### Supplementary information

Supplementary Tables

## Data Availability

The datasets presented from Experiment One and Two outlined within this paper are available on Figshare. Permission to use the datasets shared and analysed in the second half of this paper were only granted for the purposes of the independent participant data meta-analyses presented and are available from the relevant authors upon reasonable request.

## Code availability

The specific *R* scripts and code used in the analyses of Experiment One and Two are available on Figshare and are available from the corresponding author on request.
